# A Survey on the Role of Artificial Intelligence in Biobanking Studies: A Systematic Review

**DOI:** 10.3390/diagnostics12051179

**Published:** 2022-05-09

**Authors:** Gopi Battineni, Mohmmad Amran Hossain, Nalini Chintalapudi, Francesco Amenta

**Affiliations:** Clinical Research Centre, School of Medicinal and Health Products Sciences, University of Camerino, 62032 Camerino, Italy; mohammad.hossain@unicam.it (M.A.H.); nalini.chintalapudi@unicam.it (N.C.); francesco.amenta@unicam.it (F.A.)

**Keywords:** biobanks, artificial intelligence, machine learning, biomarkers, precision medicine

## Abstract

*Introduction:* In biobanks, participants’ biological samples are stored for future research. The application of artificial intelligence (AI) involves the analysis of data and the prediction of any pathological outcomes. In AI, models are used to diagnose diseases as well as classify and predict disease risks. Our research analyzed AI’s role in the development of biobanks in the healthcare industry, systematically. *Methods:* The literature search was conducted using three digital reference databases, namely PubMed, CINAHL, and WoS. Guidelines for preferred reporting elements for systematic reviews and meta-analyses (PRISMA)-2020 in conducting the systematic review were followed. The search terms included “biobanks”, “AI”, “machine learning”, and “deep learning”, as well as combinations such as “biobanks with AI”, “deep learning in the biobanking field”, and “recent advances in biobanking”. Only English-language papers were included in the study, and to assess the quality of selected works, the Newcastle–Ottawa scale (NOS) was used. The good quality range (NOS ≥ 7) is only considered for further review. *Results:* A literature analysis of the above entries resulted in 239 studies. Based on their relevance to the study’s goal, research characteristics, and NOS criteria, we included 18 articles for reviewing. In the last decade, biobanks and artificial intelligence have had a relatively large impact on the medical system. Interestingly, UK biobanks account for the highest percentage of high-quality works, followed by Qatar, South Korea, Singapore, Japan, and Denmark. *Conclusions:* Translational bioinformatics probably represent a future leader in precision medicine. AI and machine learning applications to biobanking research may contribute to the development of biobanks for the utility of health services and citizens.

## 1. Introduction

Biobanks are facilities or platforms where human biological samples are stored for future research [1,2]. Clinical data and genetic information are made available through this biorepository, which represents, also, a research resource. A biobank plays a relevant role in modern-day research, providing access to a large amount of data that can be used in various studies [2,3,4,5,6]. In the past, there was a great deal of difficulty in collecting samples or data from several different locations and using them for research. Each biobank is categorized differently. As defined by the pan-European Biobanking and Biomolecular Resources Research Infrastructure (BBMRI), population-based and disease-oriented biobanks are the most widely recognized types [3,7]. Examples of disease-oriented biobanks are those that store medical data and samples of genetic material. Alternatively, population-based biobanks are focused on the analysis and progression of acute and chronic diseases.

Computer systems that use artificial intelligence (AI) can simulate and explain human intelligence [8,9,10]. Meanwhile, machine learning (ML) and deep learning (DL) are subfields of AI, which usually gain knowledge from user experiences and improve their learning behavior over time [11,12,13]. As a field of engineering, ML entails the design, development, and evaluation of algorithmic techniques used to acquire knowledge and learn from data [14]. AI and ML models in medicine can improve patient outcomes. A holistic understanding of AI applications, opportunities, and challenges is, also, necessary from a programmatic perspective, for the ethical and sustainable implementation of AI solutions [15]. These solutions effectively address the support of decision-making, after analyzing the given user data. DL algorithms, such as neural network-based models that deal with image data, are being adopted for automatic classification and early detection of severe diseases such as cancers and neurogenerative disorders [16,17,18,19]. Explainability and causality are other AI features that contribute to more trust, fairness, and ethical responsibility. As a result, “medical AI” is an excellent AI application to study how ML can be applied for solving problems in safety and health, by using decision-making and resolving scientific problems using ML [20].

AI can be applied to all areas of healthcare to improve clinical support, including the diagnosis and prognosis of a disease. Many studies have already explained how Al is doing equal to or better than humans in healthcare industries [21,22,23,24]. Analyzing medical images, and correlating symptoms and biomarkers from electronic medical records to diagnosis and prediction of disease, are just a few examples of AI applications in the healthcare sector [25]. In biobanks, physicians find AI helpful for identifying patients who require extra care and attention, as they analyze patients’ conditions and medical exams with AI [26]. When a doctor or researcher are attempting to understand and diagnose a disease, the previous health record can be extremely helpful [26,27]. For that reason, a biobank may be the most suitable solution. Researchers and clinicians can access patient medical records from biobanks and analyze them with AI to make predictions and diagnose patients. In accessing the personal data of patients in European Union nations, the user should obey the General Data Protection Regulation (GDPR), which is a major component of human rights and privacy laws.

AI can also play a variety of roles to assist people working in biobanks [26]. AI-based computers can include and understand the information on the consent form, and can answer questions through web-based communication between Biobank members and AI. It is also critical to understand how these techniques can assist in real-time experimental works as well as to help predict patient conditions. In this paper, we describe the role of AI frameworks in the new generation of biobanking. Since big data knowledge is continuously evolving, this paper also describes how AI and ML techniques are promoting innovation and standardization in biobanking.

The remaining parts of the paper are organized as follows: Section 2 elaborates on the methods used to identify the literature, with a quality assessment of each included work; Section 3 describes the search results and characteristics of the included studies; Section 4 presents a discussion on the role and importance of biobanks; and, finally, Section 5 ends with a conclusion.

## 2. Methods

### 2.1. Search Strategy

**Figure 1:**
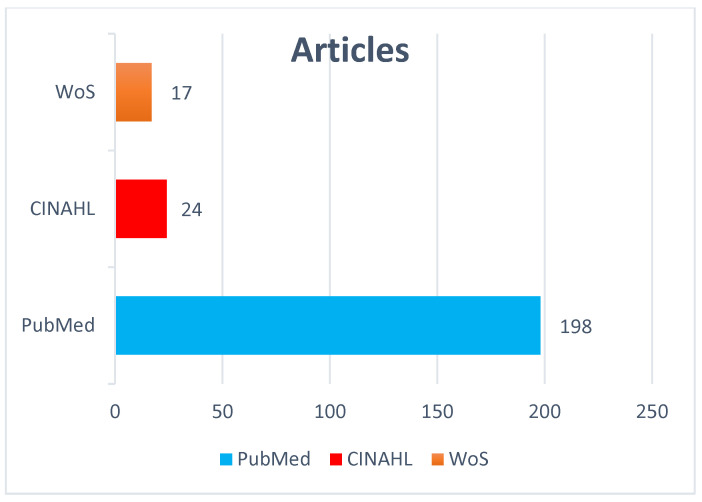
The number of papers identified is based on individual library sources.

Our literature search involved the scientific literature found in online databases such as PubMed (Medline), Cumulative Index to Nursing and Allied Health Literature (CINAHL), and Web of Science (WoS). The systematic review followed the preferred reporting items for systematic reviews and meta-analyses (PRISMA) guidelines [28]. Figure 1 shows the number of articles from selected databases. Search terms include ‘biobanks’, ‘AI or Artificial Intelligence’, ‘machine learning’, and ‘deep learning’. These are combined in search strings such as ‘biobanks and AI’, ‘deep learning in biobanks’, and ‘present advancements in biobanking’, or a string of words between them. The Boolean operator “AND” is employed in PubMed, CINAHL, and WoS to report search strings on the advances AND implications of “AI and biobanking”.

### 2.2. Selection Criteria

Based on an analysis of all retrieved articles, the authors independently evaluated the articles and drafted the list of papers considered eligible. Following this, the previously mentioned records were examined for inconsistencies, and, when one was found, the diverse opinions were discussed to reach an agreement. While determining which articles were to be included in the review, the authors read every article together to gather information helpful to achieving the end goal of the research.

Original articles published in English, as well as research using biobank datasets to identify chronic diseases, are the inclusion criteria for the study selection. Exclusion criteria were studies with review articles, books, and documents, studies with different characteristics other than inclusion criteria, studies published in non-peer-reviewed journals, and studies that did not deal with specific elements of the role of biobanking in AI.

### 2.3. Quality Evaluation

After applying inclusion and exclusion criteria, the Newcastle–Ottawa Scale (NOS) was adopted for quality checks of selected studies [29]. After applying inclusion and exclusion criteria, the Newcastle–Ottawa Scale (NOS) was adopted for quality checks of selected studies. The objective of the NOS is to assess the quality of non-randomized studies and to integrate the quality assessments into the interpretation of meta-analytical results. According to them, study quality can be divided into three categories: poor (0–4), moderate (5–6), and excellent (7–9). These scores were calculated according to study outcomes, comparability, and study groups. Different parameters that define each quality factor were also taken into account, before inclusion in the final review. Studies that reached a NOS score of at least seven (NOS ≥ 7) have been considered for further review.

## 3. Results

### 3.1. Search Outcomes

**Figure 2:**
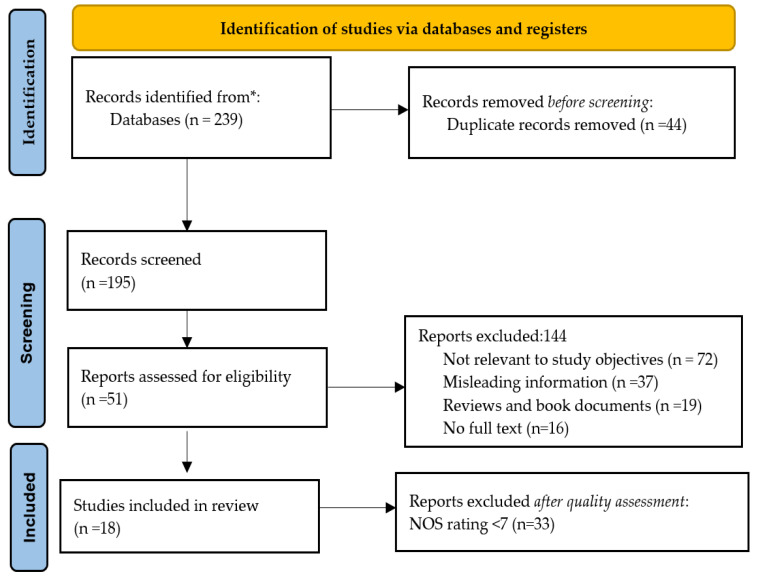
PRISMA 2020 flowchart representation for new systematic reviews, including searches of databases (* selected libraries) [30].

Our search has identified 239 items, of which 195 were retained for screening after removing duplicates (Figure 2). The following reasons led to the elimination of 128 items that were not relevant to our study objectives: 72 articles that discussed biobanks in the present world and provided general information; 37 articles that discussed information management; and 19 articles that presented book reviews. The other 16 works did not have full texts and were eliminated in the quality evaluation stage. The remaining 51 works were assessed for quality based on their content as well. In spreadsheets, all authors recorded their quality scores, following a careful application of quality criteria. Finally, 33 papers were excluded because of their low or moderate quality score (NOS < 7), and the remaining 18 were selected for final review.

### 3.2. Study Characteristics

The characteristics of each study are based on different parameters including study type, country, sample size, AI model, performance metric, and type experimental setup, which are further tabulated in Table 1.

Singapore, Japan, and Denmark are three countries that have produced studies. Two works selected from Qatar and South Korea were included in this review. The majority of the studies were based on the United Kingdom (UK) biobanks (16 out of 18). Moreover, six studies used image data from biobanks, and the rest used human participants. The input data to train the model was image type (*n* = 7), demographic data of participants (*n* = 9), and the remaining two works are applied to both image and patient data. As mentioned, AI models including both ML and deep learning were incorporated for training. Among the 18 included works, 10 works have applied supervised ML models and 6 have applied deep learning based neural network algorithms, followed by the quantification approach and quality control pipeline, respectively. Each algorithm’s performance is analyzed in terms of various metrics, such as accuracy, sensitivity (true positive rate), and receiver operating characteristics (ROC), when it comes to binary classifications. ROC analysis is widely used in medical imaging studies [31]. A ROC value of 1 indicates that a more robust classification was carried out. Five studies with binary classification presented ROC values between 0.77 and 0.91 [32,33,34,35,36]. Eight studies present their performance in terms of accuracy, ranging from 75% to 99.7% [37,38,39,40,41,42,43,44], and two studies presented sensitivity values of 69.9% [36] and 98.7% [45], respectively. Four works did not show any performance metric in their experimental outcomes [46,47,48,49]. The details of each work are discussed in the subsequent sections.

#### Biobanking Studies Associated with Image Datasets

Data from population imaging studies are used to develop and implement personalized health strategies to prevent and treat disease more effectively. The authors developed and demonstrated how to use T1-weighted MRI images from the UK biobank to predict chronological age, using convolutional neural networks (CNNs) [46]. Many clinical studies are correlated with CNN model prediction errors: ${∆}_{BrainAge}=Age{\;}_{Predicted}-Age_{True}$. The connection between ${∆}_{BrainAge}$ and image-derived phenotypes (IDPs) is also studied.

Brain images from the UK biobank are used to advance research. Automatic image processing and quality control pipelines are in place, which explains how biobank images are acquired and processed [38]. An artificial neural network called the Simple Fully Convolutional Network (SFCN) has been designed in [39]. Using T1-weighted structural MRI, they obtained very promising results, with a mean absolute error of 2.14 years and 99.5% gender classification accuracy.

Some studies proposed an automated framework by deep learning techniques for analyzing cardiac (CMR) images and determining the risk of cardiovascular disease [42], [50]. Furthermore, Alipanahi et al. [47] developed an ML model to predict vertical cup-to-disc ratio (VCDR) from color fundus photographs stored in the UK biobank, and this model successfully detected and replicated loci associated with recent VCDR genome-wide association studies.

A prospective epidemiological study obtains images of pre-symptomatic populations [51,52,53]. Many diseases can be detected early through these studies, and at-risk individuals can be identified. However, assessing the images automatically presents new challenges [54]. A few studies gathered images from three nations at the same time [34,55,56]. In [34], based on retinal images, a deep learning model was developed and validated using data from South Korea, Singapore, and the UK biobanks, to predict Coronary Artery Calcium (CAC), a validated marker of cardiovascular disease risk.

### 3.3. Applications of AI in Disease Detection with Biobanking Datasets

#### 3.3.1. Alzheimer’s Disease Detection

MRI information images can be used to classify dementia disorders (such as Alzheimer’s disease) with AI technologies and frameworks. Using the framework proposed, a sample of 500,000 AD patients’ data from the UK Biobank was successfully categorized, with an accuracy of 82.4% [37]. By using biobank information, machine learning technologies can also predict the risk of age-related macular degeneration (AMD) [32], and deep learning can uncover subnetworks that partially overlap the human brain, evaluate the relationship between social brain regions, and predict examined social traits generally, as well as predict specific aspects of social functioning, such as social isolation [57].

#### 3.3.2. Cardiovascular Diseases

Based on biobanking data from large datasets, advanced AI technologies are playing an increasingly critical role in cardiovascular disease risk prediction. As a result, one study used an auto prognosis that selects and tunes ML model features based on the auto prognosis [36].

#### 3.3.3. Chronic Diseases

AI is also being applied to analyze biomedical samples and predict risk factors for chronic diseases such as diabetes, obesity, and cancer [58,59,60]. A study analyzed 1000 patients’ data from Qatar biobanks and applied ML models to assess the risk of chronic diseases [49]. The number of risk factors for diabetes and obesity were then defined [37]. In [45], an ML model for imputing human leukocyte antigen (HLA) genotypes were developed. Data from Genome-wide association studies (GWAS) are presented in this model. They used their evaluated ML model “DEEP*HLA” to identify HLA variants associated with type 1 diabetes, independently. Another study has evaluated ML algorithms to predict aromatase inhibitor-related arthralgia (AIA), which is used to treat breast cancer patients [44]. The accuracy of the AIA prediction was 75.93%, after analyzing 695,227 single nucleotide polymorphisms (SNP) from UK biobanks [44].

Arterial hypertension is a worldwide-diffused disorder linked to several risk factors. Hypertension can be predicted and diagnosed early with the help of biobanks and artificial intelligence [40]. Adults must spend enough time walking, sleeping, and sitting to remain healthy. Monitoring these behaviors can also be accomplished using ML techniques to classify the individual’s sleep and activity levels [43].

#### 3.3.4. Disease Subtype Classification

Clinical diagnosis and treatment selection can be significantly improved by the classification of disease subtypes and correlated biomarkers [61]. Both humans and machines have difficulties in finding these subtypes in noisy, high-dimensional biomedical data. MA Schulz et al. [48] proposed a novel ML approach to naturalize disease subtype detection based on datasets of biobanks from the UK and Atlas. They introduced the classification of disease subtypes. Further, a human body MRI combined with deep neural networks can be used to provide imaged anatomy for a large-scale medical exam as well as a comprehensive medical survey [41].

#### 3.3.5. Pandemics

The ML models were able to accurately assess individual risk and track the progression of COVID-19 disease [33,62]. ML models can be used to predict the risk of COVID-19 at various stages. There is evidence that ML models can accurately predict death, hospitalization, and ICU admissions based on COVID-19 risk [33]. The authors of the study [35] developed ML models to estimate mortality risk in confirmed cases based on the COVID-19 cases obtained from the UK biobank. During the model development, they consider comorbidities such as kidney failure, urinary tract infections, pneumonia cases, and other baseline characteristics such as preexisting symptoms.

## 4. Discussion

The systematic review aims to provide a thorough analysis of the impact of AI knowledge on the health sector and to assess, systematically, the most important global biobanks. In this paper, we attempted to present how different AI techniques have been understood and applied by different authors, while remaining fair towards all sorts of biobanking datasets. ML models are viewed, based on the evidence provided, as the classification of different diseases, biomarkers, and managing of data collected from different countries’ biobanks [37,63,64,65,66].

The general observation we made is that many ML methods have been developed and are widely used to improve some analytics challenges across studies of complex human diseases. For instance, normal ageing and neurodegenerative disease cause morphological changes in the brain [46,67]. There is a subtle, non-linear, and spatially and temporally distinct effect of ageing on the brain [68,69,70]. Brain changes are frequently detected using MRI data in the clinical system. ML models can be applied to develop models that are appropriate for capturing these patterns and are responsive to changes in interest. Image analysis using deep learning algorithms performs better than manual methods in predicting diseases and diagnosing them [71,72,73]. With the use of medical images for diagnosis and prognosis, machine learning models are proving to be very efficient [32,38,39,42,46].

Medical reports are collected and stored in biobanks for future research, as such individual clinical reports are generated from biomedical images of biological samples [74,75]. The researchers can collect biobank samples and use AI techniques to automatically identify, predict, or classify risk groups in participants [76,77,78]. Traditional medical systems are struggling to diagnose chronic diseases and neurological disorders. Biobanks and AI could be supportive for clinical practice and choosing the best AI algorithms for developing disease prognosis models [79,80]. The size of the dataset, type of data, possible outcomes, and user access need to be considered when designing the model. Biobanking medical image datasets are also useful to predict and diagnose cardiovascular disease. It is reported that ML models have the satisfactory performance to predict cardiovascular disease by analyzing medical images from biobanks [34,36,42]. These models can predict the risk of developing hypertension [40] and track it to identify the physical activity of patients [43].

Regarding the data in biobanks in the future, we cannot conclude how it will be used, nor does one know what other data it can be linked to [81]. Considering that such large datasets are not easy to be handled manually, scientists are trying to develop autonomous tools to identify the hidden data patterns. The main task of finding these AI tools is to handle the larger data that is generated every day, to provide better healthcare. Biobanks are playing an important role in the transformation of personalized care, by coupling biological data with electronic health records (EHR) [66]. Big data can drive changes in perspective “from treatment to prevention”, which could allow distinguishing early variables, and, consequently, develop preventive measures. Models for anticipating health risk assessment [82], estimation of survival rates, and helpful suggestions would produce better medical services [83].

Bioinformatics is the intersection of biomedical data and informatics. Molecular and cellular technologies are creating large amounts of data, making it possible to detect and translate them into biological and clinical outcomes rapidly. Therefore, incorporating developed AI and ML technologies has the potential to provide a unique opportunity to elevate biomedical sciences. Implementing AI in biobanks can change the traditional medical system. Biobanks store participants’ biological samples and medical histories. The biobank’s data are extremely useful in the diagnostic process. Specialists evaluate treatment based on medical reports and the patient’s history.

Medical experts have classified them into high- and low-risk groups based on their medical data, as this manual procedure always takes longer and causes the diagnosis to be delayed [84]. AI can analyze medical samples in a short amount of time and predict or classify patients. The data in the biobank can be a little noisy and do not follow any specific format. The data were chosen from biobanks based on the input requirements of AI models and the expected results of this analysis. Sometimes, dealing with data necessitates more time and wisdom. It was never easy to collect data and choose the best AI model.

## 5. Conclusions

The fundamental objective of this work is to highlight the role of artificial intelligence models, which can generate a more accurate diagnosis using different kinds of data that are available in medical repositories called biobanks. Medical research and drug development are facilitated by the rapid evolution of biobanks, which can collect enormous amounts of human and non-human biological material and their related data. By developing diverse biobanks and data-sharing capabilities, researchers may be able to conduct research into personalized medicine, among other fields. Adding AI algorithms into these personalized patient data can help answer questions on genetic variation impact on human health. Translational bioinformatics can shape the future of personalized medicine. As such, this study has systematically reviewed the current trends of AI in biobanking. It is concluded that the use of AI can develop strategies for biomedical research, by analyzing the distribution and inventory statuses of the biobanks and research trends.

## Figures and Tables

**Table 1:** Summary of findings for the role of AI techniques in the management of global biobanking data.

N	Study Type	Country	Sample	Experimental Setup	Findings	Performance Metric	Ref
1	Experimental	UK	19000 T1-weighted MRI (data spilt was done for training 12,802 and testing 6885)	A three-dimensional CNN model was developed for the prediction of chronological age.	Predicted age against true age of both male and female groups from linear and nonlinear registered images.	-	[46]
2	Experimental	UK	81,830 fundus images with random seed for training and testing	An ML model was employed for predicting optic nerve head features.	Vertical cup-to-disc ratio (VCDR), a diagnostic parameter and cardinal endophenotype for glaucoma are predicted.	-	[47]
3	Experimental	UK	35,358 subjects with 32,215 Caucasians	ML models were developed for age-related macular degeneration (AMD) risk prediction.	ML models were more satisfactory than normal controls.	ROC: 0.81	[32]
4	Experimental	Japan, UK	416,846 subjects (62,387 subjects from Japan, 354,459 from the UK)	Developed DEEP*HLA deep learning models for human leukocyte antigen (HLA).	DEEP*HLA applied to subjects and succeed in linked class I and II HLA variation shared risk from those populations.	Sensitivity: 98.7%	[45]
5	Experimental	UK	Around 500,000 individuals in the age range 40 to 69	Proposed pipeline to classify Alzheimer’s disease accurately.	Modular ML models had high accuracy to detect and classify Alzheimer’s disease	Accuracy: 82.44%	[37]
6	Experimental	UK, Denmark	5594 patients	Developed and validated ML model and predicted risk of COVID-19.	ML models can predict hospital and ICU admissions risk for COVID-19 patients by using age, gender, and BMI demographic variables.	ROC: 0.80	[33]
7	Experimental	South Korea, Singapore, UK	216,152 retinal images	Five datasets from three different biobanks were used to train and validate deep learning models for coronary artery calcium (CAC) scores.	In South Korea, 6.3% of participants had cardiovascular events, and in Singapore and the UK 3.6%, and 0.7% of participants had fatal cardiovascular events, respectively.	ROC: 0.74	[34]
8	Experimental	UK	11,245 participants	Designed and validated ML model to predict mortality risk of COVID-19.	ML models are highly accurate with patient characteristics, brief medical history, symptoms, and vital signs.	ROC: 0.91	[35]
9	RCT	UK	14,503 T1-weighted structural MRI data	Data spilt was done for training 12,949 and testing 6885. Simple Fully Convolutional Network (FCN) to predict brain age.	99.5% accuracy for sex classification and brain age prediction.	Accuracy: 99.5%	[39]
10	Cross-sectional	Qatar	987 Qatar residents	Machine learning models used to predict Hypertension.	ML models are a rapid productive model to predict Hypertension.	Accuracy: 82.1%	[40]
11	Experimental	UK, ATLAS	Madelon dataset (16 classes, 50 features), fashion-MINST dataset (dimensionality: 782, sample size: 70,000), T1 brain MRI data of 10,000 participants (UKBB), 60,498 gene expressions of 8500 participants (TCGA)	Introduced an approach to discover disease subtypes: Classifier trained as healthy vs diseased to extract instance information instead of analyzing raw data.	Clustering is helpful in understanding and identification of disease subtypes.	-	[48]
12	Experimental	UK	MRI data of 32,000 participants	A neural network trained to understand various biological metrics from MRI images.	The neural network showed sturdy results to infer body measurement with MRI data.	Accuracy: 99.97%	[41]
13	Experimental	UK	20,000 subjects’ cardiac magnetic resonance (CMR) image	Fully automatic image analysis pipeline.	Experimental setup provided better significance among automation indexes and manual reference indexes. It produced similar accuracy in segmentation for humans.	Accuracy: 93%	[42]
14	Experimental	UK	423,604	ML model developed to predict cardiovascular diseases (CVD) using auto prognosis.	Auto prognosis predicted 268 more cases than the Framingham score, and also consider more predictors.	ROC: 0.77, sensitivity: 69.9%	[36]
15	Statistical	Qatar	1000	ML models and Panorama state of the art statistics methods are used to understand type 2 diabetics and obesity.	Expose the risk factor and association between diabetics and obesity to subjects.	-	[49]
16	Experimental	UK	96,220 participants	ML models to detect human sleep and activity from wrist-worn accelerometer data.	To evaluate human lifestyle and health behaviors with machine learning.	Accuracy: 87%	[43]
17	Experimental	UK	700 patients with cancer	Systematic chart review on patients with AI treatment with stage I-III BC.	This study is the primary link to a cluster of specific single nucleotide polymorphisms (SNP/gene) to aromatase inhibitor-related arthralgia (AIA) risk independent of candidate gene bias.	Accuracy: 75.93%	[44]
18	Epidemiological	UK	10,000 MRI Images	An automated processing and quality control (QC) pipeline was established.	Raw images data is converted to useful information to further research.	Accuracy: 99.1%	[38]

## Data Availability

Not applicable.

## Project Acronym

Marche Biobank (www.marchebiobank.it (accessed on 23 March 2022)).
